# Large-Scale Transcriptomic Meta-Analysis of Mouse Liver Responses to High-Fat Diet Identifies a Reproducible Core and Duration-Associated Variation

**DOI:** 10.3390/ijms27219459

**Published:** 2026-10-23

**Authors:** Oksung Chung, Ki Moon Seong, Sung Han Shim

**Affiliations:** 1 Department of Biomedical Science, College of Life Science, CHA University, Seongnam 13488, Republic of Korea; oschung@e-biogen.com; 2 R&D Center, Ebiogen Inc., Seoul 04785, Republic of Korea

**Keywords:** high-fat diet, MASLD, liver, transcriptomics, meta-analysis, reproducibility, heterogeneity, prediction interval, meta-regression, cross-species concordance

## Abstract

High-fat diet (HFD)-fed mice are widely used to investigate hepatic responses relevant to metabolic dysfunction-associated steatotic liver disease (MASLD), but reproducibility across experiments remains unclear. We performed a multilevel meta-analysis of 64 mouse liver RNA-sequencing GEO Series (GSEs) comprising 105 HFD-versus-control contrasts and compared the results with three human liver cohorts relevant to MASLD. Among 18,234 mouse genes, 6920 showed significant pooled HFD effects, whereas only 59 met all three study-defined reproducibility criteria: pooled FDR < 0.05, a 95% prediction interval excluding zero, and ≥80% of evaluable GSEs matching the pooled-effect direction (GSE-balanced directional consistency). These 59 genes constituted a high-confidence reproducible subset. Twenty-five Hallmark pathways showed significant pooled enrichment, and HFD duration was associated with 2744 gene-level effects and multiple Hallmark pathways. Mouse–human correspondence was weak, but among 10,658 one-to-one orthologs, mouse directional consistency was associated with the odds of a same-direction human effect (OR 1.075 per 10-percentage-point increase; 95% CI 1.035–1.115; *p* = 1.5 × 10^−4^). The hepatic HFD response therefore includes a small reproducible core within a broader duration-sensitive transcriptional response, while greater cross-experiment recurrence in mice was associated with modestly higher directional concordance in MASLD-relevant human liver cohorts, without constituting direct model validation.

## 1. Introduction

Metabolic dysfunction-associated steatotic liver disease (MASLD), introduced within the revised nomenclature for steatotic liver disease, is a major cause of chronic liver disease worldwide [1,2,3]. MASLD also exhibits substantial metabolic and clinical heterogeneity, including sex-related differences in disease susceptibility and progression [4]. High-fat diet (HFD)-fed mice are widely used as dietary models to investigate hepatic metabolic and molecular responses relevant to MASLD [5]. However, HFD experiments vary substantially in exposure duration, dietary composition, control-diet formulation, and animal characteristics such as sex, strain, age, and genotype [6]. Such experimental differences can influence hepatic phenotypes and the extent to which dietary models resemble human disease [7]. With liver RNA-sequencing data now available from many independent HFD studies, the reproducibility of hepatic transcriptional responses can be evaluated directly across experimental settings.

A transcriptional change observed in a single HFD experiment cannot automatically be considered a general response to HFD. Some responses may recur across diverse experimental settings, whereas others may vary across experimental conditions. We therefore evaluated average HFD effects and their reproducibility across experiments as separate but complementary features. A statistically significant pooled effect may still arise when effect magnitude, or even direction, varies substantially across experiments. Between-study heterogeneity quantifies variability beyond sampling error [8], whereas prediction intervals estimate the range of effects expected in a new independent study [9]. Directional agreement provides an additional measure of how consistently the sign of an estimated response recurs across independent experiments.

An additional question is whether responses that recur across mouse HFD experiments are also more likely to change in the same direction in human disease. Comparative transcriptomic studies have shown that dietary mouse models reproduce only selected components of human fatty liver disease and that their resemblance to human MASLD varies across models [7,10]. Limitations in the translation of mouse MASLD models to human disease therefore remain an important consideration [11]. Human MASLD is itself clinically and molecularly heterogeneous [3,12], further complicating direct cross-species comparisons. Reproducibility across mouse experiments does not necessarily imply agreement with human disease [13].

Here, we asked which hepatic transcriptional responses to HFD recur across independent experiments and which vary with exposure duration or dietary characteristics. We also asked whether mouse responses that recur more consistently across experiments are more likely to change in the same direction in human liver disease relevant to MASLD. These analyses were designed to distinguish reproducible HFD responses from those that differ across experiments and to examine whether reproducibility in mice is associated with directional agreement in human disease.

## 2. Results

### 2.1. Large-Scale Integration of Independent Mouse Hepatic HFD Transcriptomic Experiments

**Figure 1:**
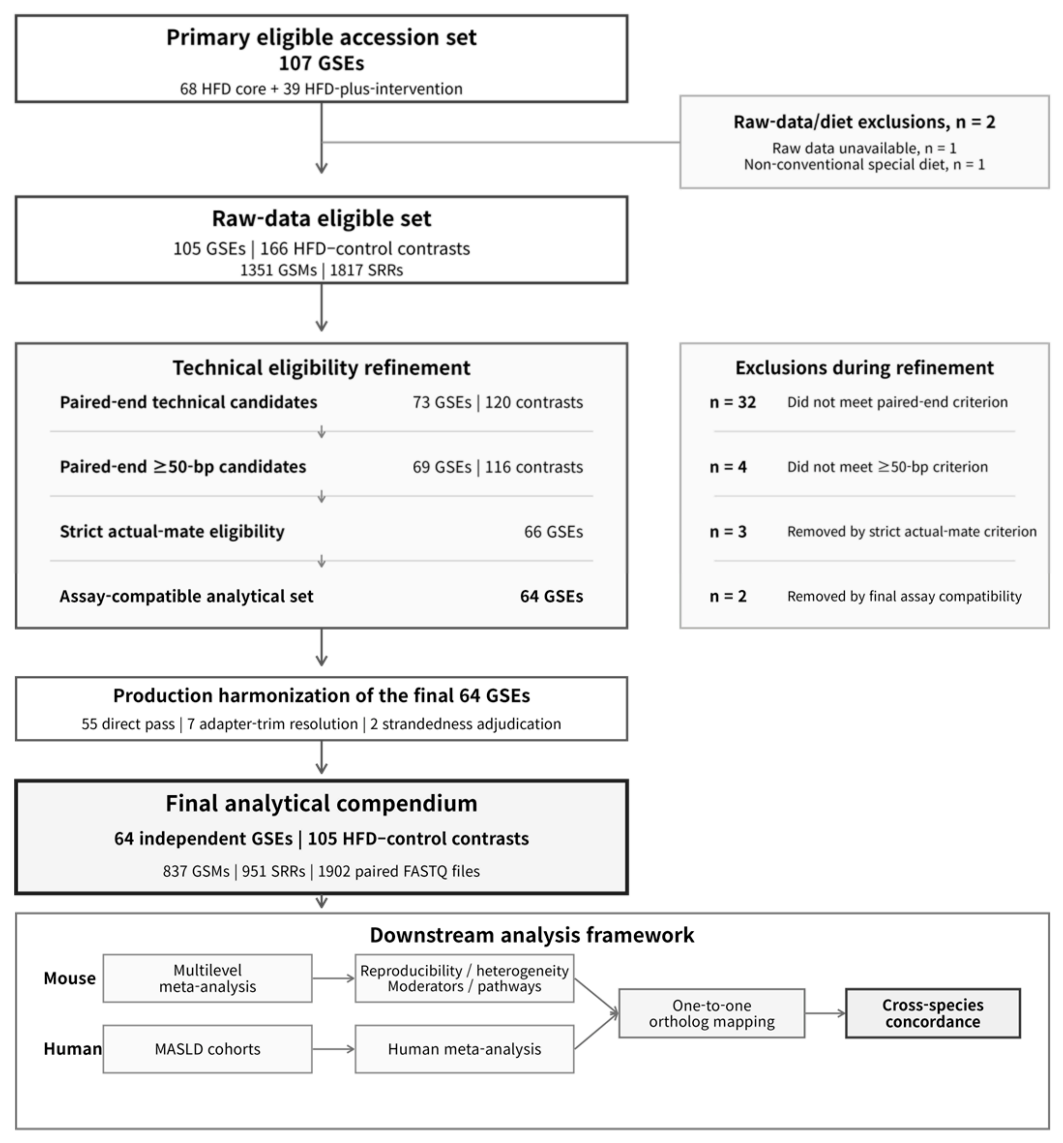
Assembly and analytical workflow of the mouse liver HFD transcriptomic compendium. The primary eligible accession set was refined through raw-data and technical eligibility review to the final analytical compendium, followed by mouse meta-analysis and cross-species integration with human MASLD.

We assembled a large compendium of publicly available mouse liver RNA-sequencing datasets comparing high-fat diet (HFD) exposure with corresponding control-diet conditions. After application of the predefined eligibility framework, sample-level reconciliation, and final accession mapping, the mouse dataset comprised 64 GSEs, 105 HFD-versus-control contrasts, 837 GEO Sample (GSM) records, and 951 Sequence Read Archive (SRR) accessions (Figure 1). Detailed characteristics of the evidence base and eligibility framework are provided in Tables S1–S3. At the GSE level, curated metadata were available for HFD duration (36/64 GSEs), dietary fat percentage (37/64), control-diet type (33/64), sex (32/64), and strain (26/64). Among annotated GSEs, the median exposure duration was 14 weeks, approximately 60% fat diets predominated (25/37 GSEs), and C57BL/6 backgrounds were common (25/26 strain-annotated GSEs). Control diets included chow (18 GSEs), low-fat diet (9), and normal diet (6), while sex representation was predominantly male and age information was inconsistently available (Table S2).

Multiple biologically distinct contrasts were retained from some GSEs under the predefined contrast rules (Table S2). Following standardized RNA-sequencing processing and contrast-level differential-expression analysis (Table S4), genes represented in at least 10 independent GSEs were eligible for the primary multilevel meta-analysis. A total of 18,234 mouse genes satisfied this criterion (Table S5).

### 2.2. Broad Average HFD Effects Contrast with More Limited Reproducibility Across Experiments

**Figure 2:**
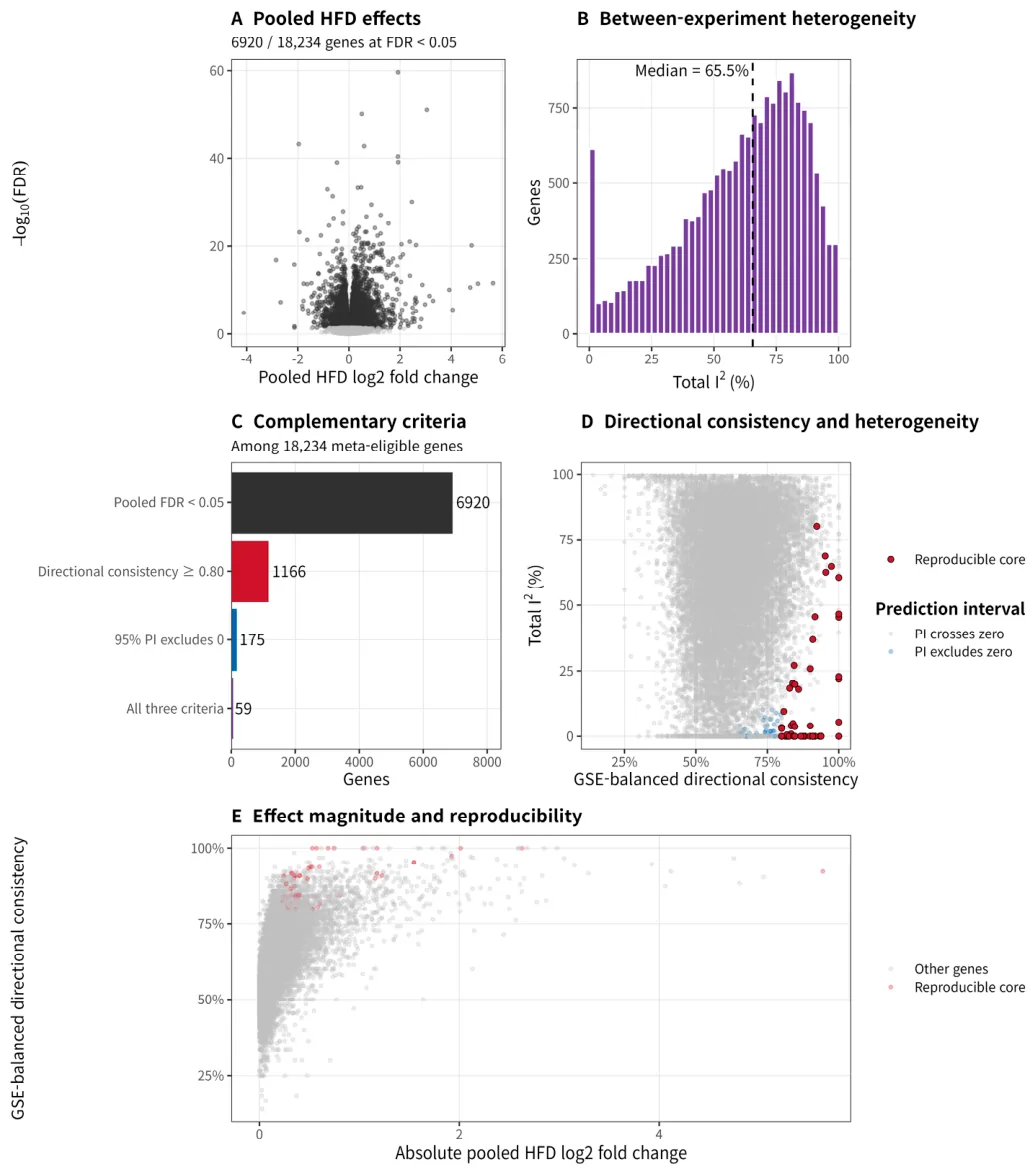
Significance, heterogeneity, and reproducibility of hepatic HFD responses. (**A**) Pooled gene-level effects. (**B**) Distribution of total I^2^. (**C**) Counts of genes meeting pooled-significance, directional-consistency, prediction-interval, and combined reproducibility criteria. (**D**) Directional consistency versus total I^2^. (**E**) Absolute pooled effect magnitude versus directional consistency.

The multilevel meta-analysis identified a broad hepatic transcriptional response to HFD. Among 18,234 genes represented in at least 10 independent GSEs, 6920 showed significant pooled effects at a Benjamini–Hochberg FDR < 0.05 (Figure 2A). However, effect sizes varied considerably across experiments, with a median total I^2^ of 65.5% across meta-analyzed genes (Figure 2B).

Measures of cross-experiment reproducibility were more restrictive. A total of 1166 genes showed GSE-balanced directional consistency ≥0.80, meaning that at least 80% of evaluable GSEs agreed with the direction of the pooled effect; 1042 of these also had pooled FDR < 0.05. Separately, 175 genes had 95% prediction intervals excluding zero, indicating that the expected effect in a new independent experiment remained on the same side of zero despite between-experiment heterogeneity; 143 of these also met the pooled FDR criterion. Sixty-three genes satisfied both reproducibility measures, irrespective of pooled significance (Table S5). These measures overlapped only partly, indicating that statistical significance alone did not capture how consistently an HFD response recurred across experiments.

### 2.3. A 59-Gene Reproducible Core and Coordinated Pathway-Level HFD Responses

**Figure 3:**
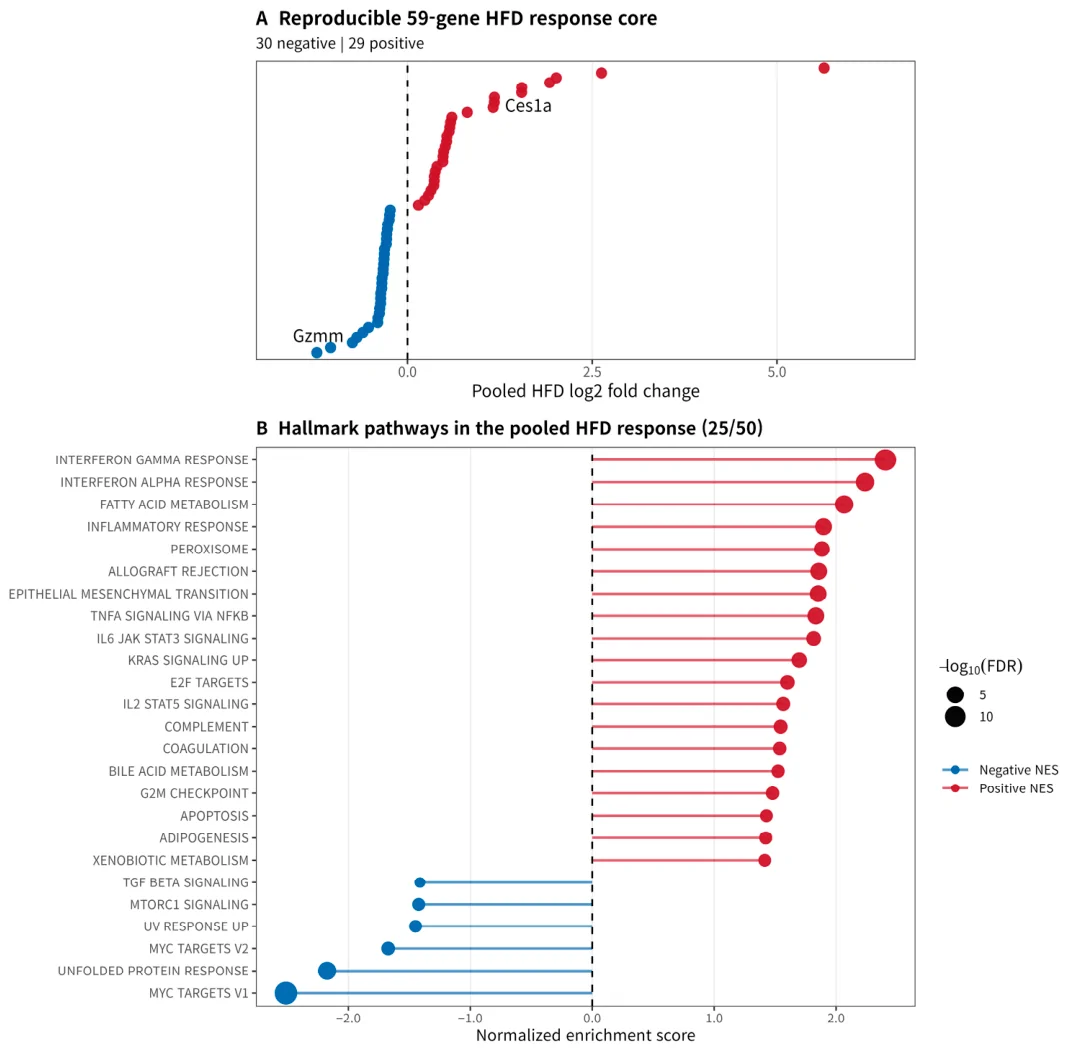
Reproducible gene-level and pathway-level HFD responses. (**A**) Pooled effects of the 59 reproducible core genes; *Ces1a* and *Gzmm* are labeled as representative annotated genes from the positive and negative reproducible core responses, respectively. (**B**) Significant Hallmark pathways in the pooled HFD response; point size represents −log10(FDR).

We next identified genes that simultaneously showed evidence of a significant average HFD effect, stability of the predicted effect across experiments, and consistent direction across independent GSEs. Fifty-nine genes met all three study-defined criteria: pooled FDR < 0.05, a 95% prediction interval excluding zero, and GSE-balanced directional consistency ≥0.80 (Figure 3A; Table S6). We defined this high-confidence subset as the 59-gene reproducible core. All 59 genes retained the direction of their pooled effect in leave-one-GSE-out refitting, providing an additional robustness check. Of these, 29 had positive pooled HFD effects and 30 had negative effects. Functionally annotated core genes represented diverse biological processes, including lipid metabolism (*Ces1a*), cytotoxic immune responses (*Gzmm*), chromatin organization (*H2ac8*, *H2bc8*, and *H4c12*), and DNA replication and maintenance (*Zranb3* and *Stn1*). However, many core genes have limited functional characterization, restricting a comprehensive functional interpretation (Table S6).

To further characterize heterogeneity, the median between-GSE and contrast-level τ^2^ values across all 18,234 genes were 0.027 and 0.048, respectively, with corresponding median I^2^ values of 19.9% and 38.7% (median total I^2^ = 65.5%; Table S5). The 59-gene core showed substantially lower median total I^2^ than pooled-significant non-core genes (approximately 0% versus 67.5%), although this difference was partly expected from the prediction-interval criterion. In a sensitivity analysis using GSE-aggregated single-level REML models, pooled effects were highly correlated with the primary estimates (Pearson r = 0.991) and all 59 core genes retained their effect directions and FDR significance.

At the pathway level, gene-set enrichment analysis of the full pooled meta-analytic gene ranking, rather than the 59-gene reproducible core, identified 25 of the 50 MSigDB Hallmark pathways as significant at FDR < 0.05 (Figure 3B; Table S7). Of these, 19 showed positive and 6 showed negative enrichment. Interferon-γ response had a normalized enrichment score (NES) of 2.405 (FDR = 9.31 × 10^−13^), and fatty acid metabolism had an NES of 2.066 (FDR = 3.00 × 10^−7^), highlighting immune and metabolic components of the pooled HFD response.

### 2.4. HFD Exposure Duration Is Associated with Variation in Thousands of Gene-Level HFD Effects

**Figure 4:**
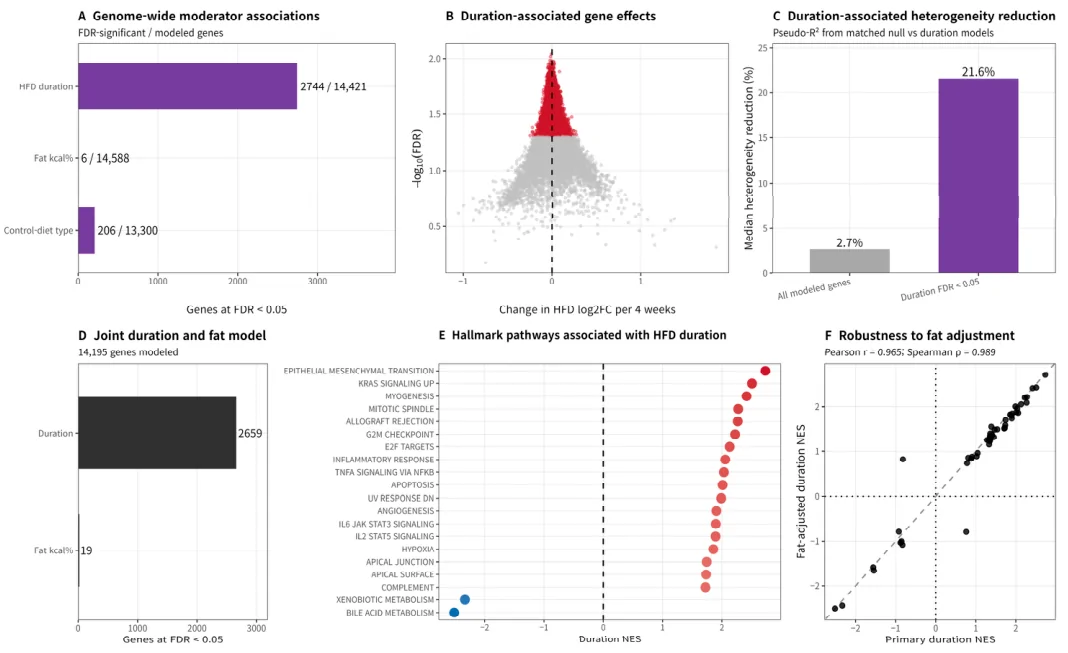
Gene- and pathway-level associations with HFD exposure duration. (**A**) Genome-wide moderator associations. (**B**) Duration-associated gene effects. (**C**) Duration-associated heterogeneity reduction. (**D**) Joint duration and fat model. (**E**) Hallmark pathways associated with HFD duration. (**F**) Concordance between primary and fat-adjusted duration NESs.

We next evaluated three predefined genome-wide moderators that met the analysis criteria: HFD exposure duration, dietary fat content expressed as kcal%, and control-diet type (Figure 4A; Table S8). Among the three moderators evaluated, HFD exposure duration was associated with the largest number of gene-level effects. Among 14,421 successfully modeled genes, 2744 exhibited significant duration-associated effects at FDR < 0.05 (Figure 4A,B; Table S9).

By comparison, dietary fat percentage showed significant moderation for only 6 of 14,588 genes when modeled per 10-percentage-point increase in energy from fat (Figure 4A; Table S10). Control-diet type, modeled categorically with CHOW as the reference, showed a significant omnibus moderator effect for 206 of 13,300 genes (Figure 4A; Table S11). Including duration as a moderator was also associated with reduced estimated residual heterogeneity, particularly among duration-associated genes. In matched models, adding duration reduced total heterogeneity by a median of approximately 2.7% across all duration-modeled genes and by 21.6% among genes with significant duration associations (Figure 4C).

Among 14,195 evaluable genes in the joint model, duration remained significant for 2659, whereas dietary fat percentage was significant for 19 (Figure 4D). In exploratory GSE-level analyses, exposure duration and dietary fat percentage showed a weak inverse correlation (Spearman ρ = −0.185; 33 GSEs). Associations were also observed among categorical study characteristics, including sex and strain (Cramér’s V = 0.622; 21 GSEs), although these estimates were descriptive and based on unevenly represented categories. At the pathway level, 29 of the 50 Hallmark pathways showed significant duration-associated enrichment. Epithelial–mesenchymal transition showed the highest positive duration-associated NES (NES = 2.734, FDR = 5.84 × 10^−19^), whereas bile acid metabolism (NES = −2.514, FDR = 3.76 × 10^−10^) and xenobiotic metabolism (NES = −2.333, FDR = 1.85 × 10^−11^) showed negative associations with duration. After adjustment for dietary fat percentage, 48 of 50 Hallmark pathways retained the same NES direction, the primary and adjusted NESs were correlated (Pearson r = 0.965; Spearman ρ = 0.989), and 25 pathways remained significant at FDR < 0.05 (Figure 4E,F; Table S12).

### 2.5. More Reproducible Mouse Responses Are More Often Directionally Concordant in MASLD-Relevant Human Liver Cohorts

To assess whether reproducible mouse HFD responses were also more likely to change in the same direction in human disease, we analyzed three independent human liver cohorts relevant to MASLD (GSE126848, GSE130970, and GSE135251) (Tables S13 and S14). The cohorts differed in clinical composition: GSE126848 included 15 NAFL and 16 NASH cases, GSE130970 covered a broad range of NAFLD activity scores and fibrosis stages, and GSE135251 included NAFLD cases across different fibrosis stages. All three cohorts used liver biopsy specimens, but detailed clinical covariates were not consistently harmonized across studies (Table S13). Genes with estimable effects in all three cohorts yielded 12,477 genes for the human meta-analysis (Table S15). Across these 12,477 genes, the median between-cohort heterogeneity variance (τ^2^) was 0.084, and the median I^2^ was 83.2%, indicating substantial heterogeneity among the human cohorts (Table S15). High-confidence one-to-one ortholog mapping identified 10,658 genes represented in both the mouse and human analyses (Table S16).

**Figure 5:**
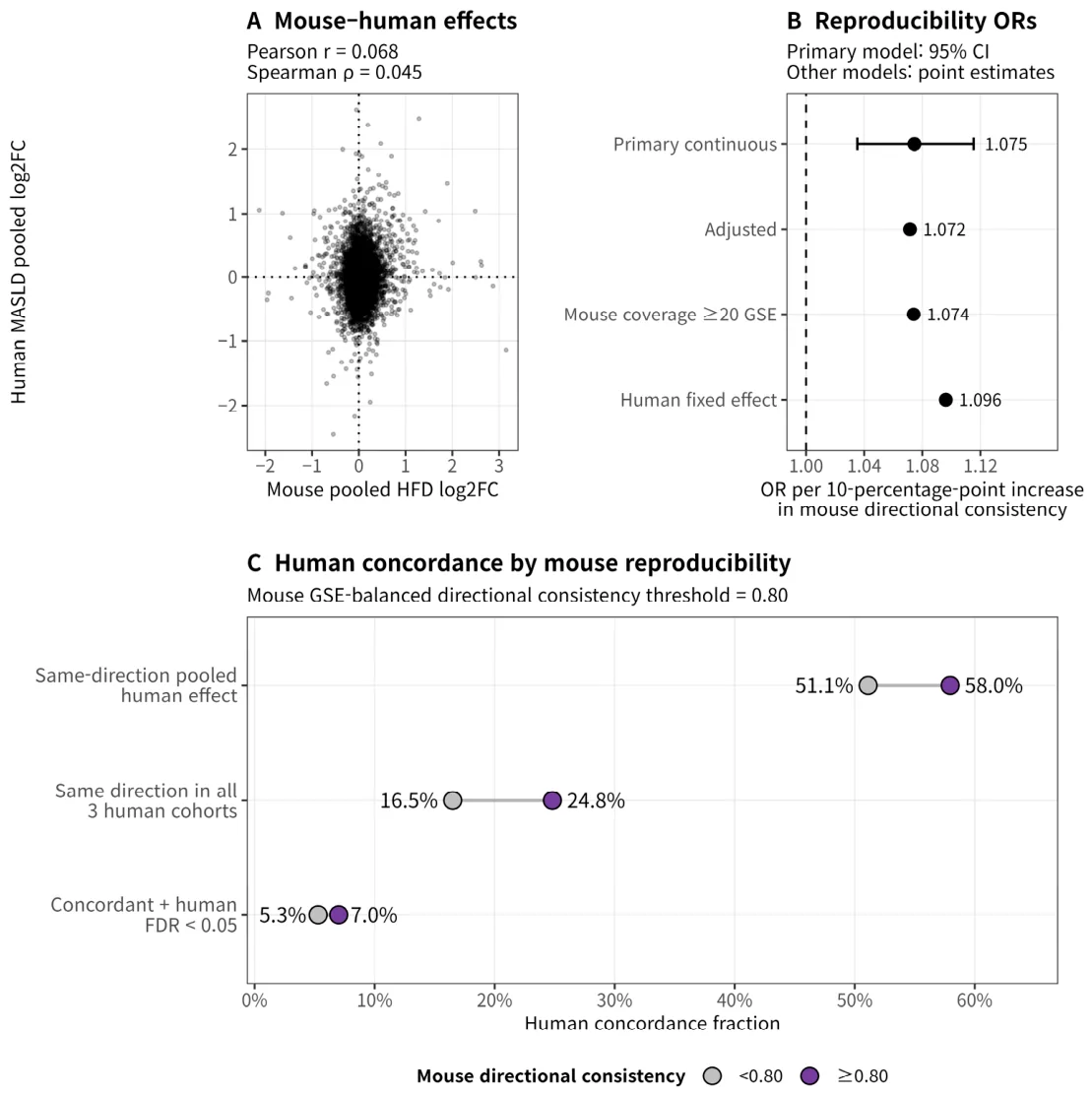
Mouse cross-experiment reproducibility and directional concordance in MASLD-relevant human liver cohorts. (**A**) Global gene-level correspondence between pooled mouse HFD and human MASLD effects. (**B**) Association between mouse GSE-balanced directional consistency and same-direction human effects in the primary and sensitivity analyses; the 95% confidence interval is shown for the primary model. (**C**) Fractions of genes showing pooled directional concordance, concordant directions across all three human cohorts, or concordant effects reaching human FDR < 0.05, stratified by mouse GSE-balanced directional consistency <0.80 or ≥ 0.80.

At the global level, pooled mouse HFD and human MASLD effects showed only weak correspondence (Pearson r = 0.068; Spearman ρ = 0.045) (Figure 5A), indicating limited overall gene-level concordance between the mouse HFD response and human MASLD. We therefore asked a more focused question: whether mouse responses that recurred more consistently across independent experiments were more likely to show the same direction of change in human disease.

The primary logistic regression included 10,658 orthologous genes, of which 5484 (51.5%) showed concordant pooled mouse and human effect directions. Each 10-percentage-point increase in GSE-balanced directional consistency was associated with a 7.5% increase in the odds of directional concordance (OR = 1.075, 95% CI 1.035–1.115; *p* = 1.5 × 10^−4^) (Figure 5B; Table S17). For illustration, a 20-percentage-point difference corresponds to an estimated OR of 1.16 under the fitted logistic model; this represents an association across genes rather than a prediction of individual gene behavior. This association remained statistically significant after adjustment for mouse effect magnitude and study coverage, restriction to genes represented in at least 20 mouse GSEs, and use of fixed-effect estimates for the human analysis. In exploratory leave-one-human-cohort-out analyses using fixed-effect estimates from the remaining two cohorts, the association remained positive (ORs = 1.069–1.116; all *p* < 0.001).

Consistent with this association, genes with higher mouse reproducibility also showed higher observed fractions of directional concordance in the human cohorts. Among 528 genes with GSE-balanced directional consistency ≥0.80, 306 (58.0%) showed same-direction human effects compared with 5178 of 10,130 genes (51.1%) below this threshold. The same pattern was observed using stricter concordance definitions: genes with mouse directional consistency ≥0.80 more often showed concordant effects reaching human FDR < 0.05 (7.0% vs. 5.3%) and concordant directions across all three human cohorts (24.8% vs. 16.5%) (Figure 5C).

## 3. Discussion

Among more than 18,000 hepatic genes, only 59 formed a reproducible HFD-response core; the broader transcriptomic response varied with exposure duration, and cross-experiment reproducibility was associated with directional concordance with human MASLD. The hepatic transcriptional response to HFD was therefore not uniform across experiments: a small reproducible component coexisted with a much broader response that varied across experiments. Despite substantial gene-level heterogeneity, coordinated immune and metabolic responses were evident at the pathway level. Among the moderators examined, exposure duration was associated with variation in the largest number of gene-level HFD effects.

### 3.1. Reproducibility-Based Resolution of the Hepatic HFD Response

The mouse meta-analysis revealed a marked distinction between average statistical significance and cross-experiment reproducibility. Of 18,234 genes, 6920 showed significant pooled HFD effects, yet only 59 satisfied all three study-defined reproducibility criteria and retained their pooled-effect direction in every leave-one-GSE-out analysis. Prediction intervals and GSE-balanced directional consistency therefore identified a subset of responses whose expected effect and direction remained stable across independent experiments—beyond what pooled significance alone could establish [14].

The 59-gene reproducible core, however, does not represent the full biological response to HFD. At the pathway level, the broader pooled transcriptome showed coordinated immune and metabolic responses, including interferon-γ response and fatty acid metabolism, despite substantial gene-level heterogeneity. The data therefore separate the hepatic HFD response into two broad components: a small core that recurs across experiments and a much larger set of responses that varies across experimental conditions. Genes outside the core may remain biologically relevant but fail to meet the criteria because of experimental heterogeneity, limited GSE coverage, technical variability, or insufficient statistical power. Distinguishing average effects from cross-experiment recurrence may therefore improve how transcriptomic evidence from HFD models is interpreted [15,16].

### 3.2. Exposure Duration Is Associated with Gene- and Pathway-Level Differences Across HFD Studies

Exposure duration showed the broadest association with variation in hepatic transcriptional responses among the predefined experimental moderators. Thousands of genes showed duration-associated changes, and 2659 remained significant when dietary fat percentage was included in the same model. Importantly, duration did not reduce heterogeneity uniformly across all genes: the median reduction was modest across all duration-modeled genes but substantially larger among genes with significant duration associations. Thus, the effect of exposure duration was concentrated in a subset of HFD-responsive genes rather than distributed uniformly across the transcriptome.

Duration-associated effects were also evident at the Hallmark pathway level, indicating that exposure time is linked to coordinated shifts in hepatic transcriptional programs. Remodeling- and inflammation-related programs—including epithelial–mesenchymal transition, inflammatory response, and TNFα signaling via NF-κB—showed positive duration-associated enrichment, whereas bile acid and xenobiotic metabolism showed negative duration-associated enrichment. Notably, bile acid and xenobiotic metabolism were positively enriched in the pooled HFD response but negatively associated with duration. This contrast indicates that longer exposure is not simply associated with a stronger version of the same HFD response, but with a shift in its relative pathway composition. Previous time-course studies have shown that metabolic, inflammatory, and fibrotic responses develop along distinct temporal trajectories during dietary exposure [17,18]. Our cross-study analysis similarly showed that hepatic gene-expression profiles differed across experiments with different HFD durations. Exposure duration therefore distinguished not only individual gene responses but also the relative balance of metabolic, inflammatory, and remodeling programs.

By contrast, dietary fat percentage showed comparatively limited gene-level moderation. This finding should not be interpreted as evidence that dietary composition is unimportant. Studies with available fat-content metadata were concentrated around commonly used high-fat formulations, particularly diets providing approximately 60% of energy from fat, limiting the effective range of this moderator. Moreover, fat kcal% does not capture differences in fat source, carbohydrate and protein composition, cholesterol supplementation, feeding regimen, control-diet formulation, or actual energy intake; thus, adjustment for fat kcal% alone cannot exclude dietary confounding of the duration associations. Control-diet type also contributed to systematic variation, although control categories were unevenly represented and most significant pairwise signals involved LFD-versus-CHOW comparisons. Among the moderators examined, duration was associated with the largest number of gene-level differences, whereas other dietary features could not be evaluated with comparable statistical coverage. However, these duration associations should not be interpreted causally, as exposure duration may be confounded by other experimental characteristics, including animal age, sex, strain, dietary composition, and study design.

### 3.3. Mouse Reproducibility and Human Directional Agreement

Overall gene-level correspondence between mouse HFD and MASLD-relevant human liver cohorts was weak, consistent with previous studies showing that dietary mouse models capture selected rather than comprehensive components of the human disease transcriptome [7,10,11]. 

Several biological and experimental factors may contribute to the weak mouse–human correspondence and the modest association observed here. Human MASLD involves complex interactions among hepatic cell populations and extrahepatic organs, including gut–liver signaling and potentially spleen–liver immune crosstalk, which cannot be fully captured by bulk liver transcriptomic comparisons alone [19,20]. Human MASLD also encompasses steatohepatitis, fibrosis, chronic inflammation, and cardiometabolic complications that may not be fully reproduced by individual HFD protocols [3,7]. Differences in genetic background, comorbidities, medication exposure, and hepatic cellular composition may further contribute to heterogeneity among human cohorts [3,21]. In addition, mouse HFD studies differ in exposure duration and dietary formulation, including fat and carbohydrate sources and cholesterol supplementation [7]. Although these factors provide plausible explanations for the limited cross-species correspondence, their individual contributions to the observed association were not directly evaluated.

Nevertheless, genes with GSE-balanced directional consistency ≥0.80 showed same-direction human effects more often than genes below this threshold (58.0% vs. 51.1%), and greater directional consistency remained associated with human concordance when modeled as a continuous gene-level property. Similar patterns were observed under stricter concordance definitions requiring statistical significance in the human meta-analysis or directional agreement across all three human cohorts.

Despite the weak global correlation, more reproducible mouse responses were somewhat more likely to show the same direction of change in human disease. Accordingly, the cross-species analysis evaluated reproducibility across the broader ortholog-mapped transcriptome rather than treating the 59-gene reproducible core as a direct human validation set. Thus, greater recurrence across mouse experiments was associated with modestly higher directional concordance in human liver cohorts, but this association does not establish predictive utility or direct validation of the mouse model [13].

### 3.4. Strengths and Limitations

A major strength of this study is the integration of a large and experimentally diverse collection of mouse liver HFD transcriptomic datasets using the same processing and meta-analytic procedures. The multilevel meta-analysis retained biologically distinct contrasts while accounting for their shared study origin, while pooled effects, prediction intervals, and GSE-balanced directional consistency allowed average effects, expected effects in new experiments, and directional recurrence across GSEs to be evaluated separately. Moderator and pathway analyses further linked these responses to experimental variables and biological pathways, while comparison with independent human liver cohorts relevant to MASLD provided a focused assessment of cross-species directional concordance.

Several limitations should be considered. Because the analysis was restricted to publicly available GEO datasets with accessible raw sequencing data and compatible sequencing configurations, studies with null or unexpected findings may be underrepresented. Although dataset selection was independent of differential-expression results, publication and data-availability biases cannot be excluded and may limit the generalizability of the findings. Public HFD datasets vary in experimental design and metadata completeness, and the moderator analyses therefore describe cross-study associations rather than causal effects. Some potentially important experimental factors, including sex, strain, age, and genotype, could not be reliably evaluated as additional genome-wide moderators because of incomplete, unevenly represented, or insufficiently harmonized metadata. The predominance of male mice, C57BL/6 backgrounds, and approximately 60% fat diets among annotated studies may limit the generalizability of our findings to less-represented experimental settings. Although the multilevel model partitioned heterogeneity into between-GSE and contrast-level components, the specific biological and technical sources of this variation could not be fully identified. Despite expression filtering and effect-level quality control, unshrunken log2 fold changes may remain unstable for lowly expressed genes. The 59-gene reproducible core reflects a study-defined operational definition rather than a complete catalog of biologically relevant HFD responses. Bulk liver transcriptomes also reflect both transcriptional regulation and differences in cellular composition [21]. Accordingly, the 59-gene core and major pathway findings may partly reflect changes in hepatocyte, macrophage, or stellate-cell abundance rather than exclusively cell-intrinsic transcriptional responses. These contributions could not be distinguished using bulk RNA-sequencing data alone. Finally, the human comparison involved three heterogeneous liver cohorts relevant to MASLD and was designed to assess directional concordance rather than to validate the mouse HFD response as a comprehensive model of human disease. The small control groups in two human cohorts may have reduced the precision of effect estimates, although cohort-exclusion analyses supported the direction of the observed association. Differences in disease severity and incomplete harmonization of clinical characteristics—including age, sex, BMI, diabetes status, and fibrosis stage—may also contribute to the weak overall mouse–human correspondence.

## 4. Materials and Methods

### 4.1. Study Design and Mouse HFD Compendium

The NCBI Gene Expression Omnibus (GEO) DataSets database was searched using NCBI EDirect (version 12.0). Eight broad and eight restrictive Boolean queries combined *Mus musculus*, liver or hepatic tissue terms, HFD-related keywords (including high-fat diet, HFD, and diet-induced obesity), and high-throughput sequencing dataset filters. The restrictive searches additionally used sample-source or description fields and sample-count filters. The initial GEO search was conducted on 15 June 2026, and the archived searches were completed on 13 August 2026. Complete search expressions and scripts are provided in the accompanying analysis code repository. Eligible datasets included *Mus musculus* whole-liver bulk RNA-sequencing experiments with wild-type animals or an extractable wild-type/control subset; a conventional HFD condition with an appropriate chow, standard-diet, normal-diet, or low-fat-diet control; at least three biological replicates per group; and publicly accessible raw sequencing data. Comparisons involving concurrent disease-inducing interventions, engineered disease genotypes, or incompatible sequencing configurations were excluded. Eligibility was determined independently of differential-expression magnitude, concordance with other studies, pathway enrichment, or downstream meta-analysis results. Detailed accession-level screening, technical refinement, and contrast definitions are provided in Figure 1 and Tables S1–S3.

### 4.2. Mouse RNA-Sequencing Processing and Differential Expression

Mouse sequencing reads were aligned to the GRCm39 reference genome with GENCODE M39 annotation [22] using STAR version 2.7.10b [23], and gene-level counts were generated with featureCounts from Subread version 2.0.6 [24]. Mapping quality, strandedness, and technical-run consistency were reviewed before statistical analysis. Within each contrast, genes with at least 10 raw counts in at least three biological samples were retained and analyzed with DESeq2 version 1.42.1 [25] using group as the sole design factor. All R-based statistical analyses were performed using R version 4.3.1. Independent filtering was disabled, and automatic count replacement for Cook’s-distance outliers was not applied. The primary meta-analytic effect was the unshrunken log2 fold change, with sampling variance calculated from the squared standard error. Non-finite effect estimates or standard errors, non-positive standard errors, and Cook’s-distance outliers were excluded from downstream meta-analysis.

### 4.3. Multilevel Mouse Meta-Analysis and Reproducibility

Genes with estimable effects from at least 10 independent GSEs were included in the primary mouse meta-analysis. Multilevel random-effects models were fitted using rma.mv in metafor version 4.8.0 [26] with restricted maximum likelihood (REML) estimation. Random effects were specified for GSE and individual contrast identifiers, allowing multiple biologically distinct contrasts from the same GSE to be retained while accounting for their shared study origin. Ten GSEs contributed multiple contrasts; for example, GSE278920 provided 12 comparisons stratified by mouse strain and tissue-collection time (Tables S2 and S3). Examination of sample-to-contrast assignments confirmed that no GSM or SRR accession was shared across the 105 contrasts. Although GSE-level random effects accounted for study-level clustering, sampling-error correlations were not explicitly modeled, and unrecorded biological or technical dependencies could not be excluded. Pooled-effect inference used z-based tests, and *p* values were adjusted across genes using the Benjamini–Hochberg procedure.

Heterogeneity was partitioned into between-GSE and additional between-contrast variance components, which were combined to obtain total heterogeneity. Total I^2^ summarized the proportion of observed variation attributable to heterogeneity beyond sampling error. A 95% prediction interval was calculated as the pooled effect ±1.96 times the square root of the sum of the squared pooled-effect standard error, between-GSE variance (τ^2^_GSE), and contrast-level variance (τ^2^_contrast), representing the expected range of effects in a new contrast from an independent GSE.

Cross-experiment reproducibility was additionally assessed using GSE-balanced directional consistency. For each gene, multiple contrasts within a GSE were first combined into a single inverse-variance-weighted effect estimate using weights of 1/SE^2^. GSE-balanced directional consistency was calculated as the proportion of evaluable GSEs whose aggregated effects agreed in sign with the pooled meta-analytic effect. Each evaluable GSE contributed one directional vote, regardless of the number of contrasts within that GSE. The reproducible core was defined by three study-defined criteria: pooled FDR < 0.05, a 95% prediction interval excluding zero, and GSE-balanced directional consistency ≥0.80. The first two use conventional statistical levels, whereas 0.80 is an investigator-defined threshold intended to require substantial directional agreement while allowing limited heterogeneity. Leave-one-GSE-out refitting was used as an additional robustness check, with directional robustness defined as preservation of the pooled-effect direction after omission of each individual GSE. As a sensitivity analysis, contrast-level effects were aggregated within each GSE using inverse-variance weighting, followed by single-level random-effects meta-analysis with REML estimation.

### 4.4. Experimental Moderator Analyses

Three variables were predefined as primary genome-wide moderators: HFD exposure duration, HFD fat content expressed as kcal%, and control-diet type. Sex and strain were not included as additional genome-wide moderators because of uneven representation across independent GSEs, whereas age and genotype information could not be sufficiently harmonized for reliable cross-study comparisons. Meta-regression used the same multilevel REML structure as the primary analysis. Continuous moderator analyses required at least 20 independent GSEs and at least two distinct moderator values. Control-diet analyses additionally required at least five GSEs in each category, with the omnibus moderator test treated as primary. Missing moderator values were not imputed. Each moderator analysis included only eligible contrasts with available annotations for the corresponding moderator, whereas the joint duration-plus-fat model required both moderator values to be available. Continuous moderators were centered and rescaled but were not standardized to unit variance. Duration was centered at 12 weeks and scaled per additional 4 weeks, while HFD fat content was centered at 60% of energy from fat and scaled per additional 10 percentage points. Control-diet type was modeled categorically as CHOW, low-fat diet (LFD), or normal diet, with CHOW as the reference. Benjamini–Hochberg correction was applied separately within each genome-wide moderator analysis. No additional global FDR correction was applied across the separate moderator analyses or across distinct gene-level and pathway-level analyses; thus, reported FDR values reflect multiple-testing adjustment within each respective analysis. A joint duration-plus-fat model was used as a sensitivity analysis among genes evaluable for both continuous moderators. To assess the extent to which duration accounted for heterogeneity, total heterogeneity from the duration model was compared with that from a matched null model and the percentage reduction was summarized descriptively. Potential associations among experimental moderators were explored using GSE-level summaries of curated metadata. Continuous variables were summarized by their within-GSE medians, and categorical comparisons included GSEs with a single recorded category per variable. Spearman correlations and Cramér’s V were used for descriptive assessment.

### 4.5. Mouse Hallmark Pathway Analysis

Mouse pathway analyses used the native mouse MSigDB 2025.1.Mm Hallmark collection [27]. Preranked gene-set enrichment analysis [28] was performed using fgseaMultilevel in fgsea version 1.28.0 [29], with gene-set sizes between 15 and 500. Within each Hallmark pathway analysis (pooled HFD response, duration-associated response, and fat-adjusted duration-associated response), enrichment *p* values were adjusted across the tested pathways using the Benjamini–Hochberg procedure. Pooled-response enrichment ranked genes by the z statistic from the primary multilevel meta-analysis, whereas duration-associated enrichment used the duration meta-regression statistic. Adjustment for HFD fat content was evaluated as a sensitivity analysis. The ranked gene universe comprised all genes with estimable statistics in the corresponding analyses (18,234 for pooled HFD effects, 14,421 for duration, and 14,195 for fat-adjusted duration). Hallmark gene sets were evaluated using their overlap with the respective ranked universe, rather than the full reference database or only the 59-gene reproducible core.

### 4.6. Human MASLD Transcriptomic Meta-Analysis

Three independent human liver cohorts originally characterized under the NAFLD/NASH nomenclature were analyzed as disease cohorts relevant to current MASLD: GSE126848 (31 cases, 26 controls), GSE130970 (72 cases, 6 controls), and GSE135251 (206 cases, 10 controls). Primary case/control assignments were defined from the source cohort annotations and are detailed in Table S13. GEO data were retrieved and handled using GEOquery version 2.70.0 [30].

Cohort-level differential-expression analysis was performed using edgeR version 4.0.16 [31] and limma version 3.58.1 [32]. Low-expression genes were removed using filterByExpr, libraries were normalized using the trimmed mean of M-values (TMM) method [33], and expression values were transformed using voom [34]. Case–control effects were estimated with linear models and empirical Bayes moderation. Genes with estimable effects in all three cohorts were combined using REML random-effects models with rma.uni in metafor [26], with fixed-effect models evaluated as a sensitivity analysis. Meta-analysis *p* values were adjusted using the Benjamini–Hochberg procedure.

### 4.7. Mouse-Human Ortholog Mapping and Cross-Species Concordance

Mouse and human results were linked using Ensembl Compara release 116 [35]. Unique one-to-one orthologs with orthology confidence equal to 1 were retained and intersected with genes represented in both species to define the translational gene set. Global cross-species correspondence was summarized using Pearson and Spearman correlations between pooled mouse HFD and human effects.

The primary cross-species endpoint was directional concordance, defined as agreement in sign between pooled mouse and human effects. Its association with mouse GSE-balanced directional consistency was evaluated by logistic regression, with directional consistency scaled per 10-percentage-point increase. Each orthologous gene contributed one binary observation based on the directional agreement between pooled mouse and human effects. Because human cohort-specific effects were combined before logistic regression, no gene- or cohort-level clustering adjustment was applied; correlations among different genes were not explicitly modeled. An adjusted model additionally included absolute mouse pooled log2 fold change and the log10-transformed number of contributing mouse GSEs. Sensitivity analyses used human fixed-effect estimates and restriction to genes represented in at least 20 mouse GSEs. Additional leave-one-human-cohort-out sensitivity analyses combined the remaining two cohorts using inverse-variance fixed-effect weighting and refitted the directional-concordance logistic model. For descriptive comparisons, genes were also stratified by mouse directional consistency <0.80 or ≥ 0.80, and human concordance was summarized by pooled directional agreement, concordant pooled human effects reaching FDR < 0.05, and concordant direction across all three human cohorts.

## 5. Conclusions

Across 64 mouse liver HFD transcriptomic datasets, 59 genes met all three study-defined reproducibility criteria and defined a reproducible hepatic HFD-response core. Beyond this core, exposure duration was associated with variation in thousands of gene-level HFD effects and with changes across multiple Hallmark pathways. Despite weak global mouse–human correspondence, mouse responses that recurred more consistently across experiments were somewhat more likely to show same-direction effects in MASLD-relevant human liver cohorts; this association does not constitute direct validation of the mouse model. Together, these results distinguish a small set of recurring hepatic HFD responses from a broader transcriptional response that varies across experiments, with exposure duration showing the broadest association among the experimental moderators examined.

## Data Availability

All mouse and human transcriptomic datasets analyzed in this study are publicly available from the NCBI Gene Expression Omnibus and Sequence Read Archive under the accession numbers reported in the Supplementary Tables. Key processed analysis outputs and supporting data are provided in the Supplementary Materials. Frozen analytical inputs, derived results, sensitivity analysis outputs, and reference annotations are available on Zenodo at https://doi.org/10.5281/zenodo.23355111 (accessed on 21 September 2026). The analysis code and reproducibility documentation are available on GitHub at https://github.com/okokookkk/HFD-Liver-Transcriptome-MetaAnalysis (accessed on 21 September 2026) and archived on Zenodo at https://doi.org/10.5281/zenodo.23358779 (accessed on 21 September 2026).

## Supplementary Materials

The following supporting information can be downloaded at: https://www.mdpi.com/article/10.3390/ijms27219459/s1.

## Abbreviations

The following abbreviations are used in this manuscript:

HFD	High-Fat Diet
MASLD	Metabolic Dysfunction-Associated Steatotic Liver Disease
GEO	Gene Expression Omnibus
GSE	GEO Series
GSM	GEO Sample
SRR	Sequence Read Archive Run
FDR	False Discovery Rate
LFD	Low-Fat Diet
REML	Restricted Maximum Likelihood
NES	Normalized Enrichment Score
TMM	Trimmed Mean of M-values
MSigDB	Molecular Signatures Database

## References

[1] Younossi Z.M., Golabi P., Paik J.M., Henry A., Van Dongen C., Henry L. (2023). The global epidemiology of nonalcoholic fatty liver disease (NAFLD) and nonalcoholic steatohepatitis (NASH): A systematic review. Hepatology.

[2] Rinella M.E., Lazarus J.V., Ratziu V., Francque S.M., Sanyal A.J., Kanwal F., Romero D., Abdelmalek M.F., Anstee Q.M., Arab J.P. (2023). A multisociety Delphi consensus statement on new fatty liver disease nomenclature. Hepatology.

[3] European Association for the Study of the Liver (EASL), European Association for the Study of Diabetes (EASD), European Association for the Study of Obesity (EASO) (2024). EASL-EASD-EASO Clinical Practice Guidelines on the management of metabolic dysfunction-associated steatotic liver disease (MASLD). J. Hepatol..

[4] Jamalinia M., Saeian S., Nikkhoo N., Nazerian A., Lankarani K.B. (2025). Sex and gender differences in MASLD: Pathophysiological mechanisms, clinical implications, and future directions. Metab. Target Organ Damage.

[5] Im Y.R., Hunter H., de Gracia Hahn D., Duret A., Cheah Q., Dong J., Fairey M., Hjalmarsson C., Li A., Lim H.K. (2021). A systematic review of animal models of NAFLD finds high-fat, high-fructose diets most closely resemble human NAFLD. Hepatology.

[6] Montero-Vallejo R., Maya-Miles D., Ampuero J., Martín F., Romero-Gómez M., Gallego-Durán R. (2024). Novel insights into metabolic-associated steatotic liver disease preclinical models. Liver Int..

[7] Vacca M., Kamzolas I., Harder L.M., Oakley F., Trautwein C., Hatting M., Ross T., Bernardo B., Oldenburger A., Hjuler S.T. (2024). An unbiased ranking of murine dietary models based on their proximity to human metabolic dysfunction-associated steatotic liver disease (MASLD). Nat. Metab..

[8] Higgins J.P.T., Thompson S.G., Spiegelhalter D.J. (2009). A re-evaluation of random-effects meta-analysis. J. R. Stat. Soc. Ser. A Stat. Soc..

[9] IntHout J., Ioannidis J.P.A., Rovers M.M., Goeman J.J. (2016). Plea for routinely presenting prediction intervals in meta-analysis. BMJ Open.

[10] Teufel A., Itzel T., Erhart W., Brosch M., Wang X.Y., Kim Y.O., von Schönfels W., Herrmann A., Brückner S., Stickel F. (2016). Comparison of gene expression patterns between mouse models of nonalcoholic fatty liver disease and liver tissues from patients. Gastroenterology.

[11] Gunes A., Estall J.L. (2024). Is MASLD lost in translation in mice?. Trends Endocrinol. Metab..

[12] Kendall T.J., Jimenez-Ramos M., Turner F., Ramachandran P., Minnier J., McColgan M.D., Alam M., Ellis H., Dunbar D.R., Kohnen G. (2023). An integrated gene-to-outcome multimodal database for metabolic dysfunction-associated steatotic liver disease. Nat. Med..

[13] Dirnagl U., Duda G.N., Grainger D.W., Reinke P., Roubenoff R. (2022). Reproducibility, relevance and reliability as barriers to efficient and credible biomedical technology translation. Adv. Drug Deliv. Rev..

[14] Borenstein M. (2024). Avoiding common mistakes in meta-analysis: Understanding the distinct roles of Q, I-squared, tau-squared, and the prediction interval in reporting heterogeneity. Res. Synth. Methods.

[15] Sweeney T.E., Haynes W.A., Vallania F., Ioannidis J.P., Khatri P. (2017). Methods to increase reproducibility in differential gene expression via meta-analysis. Nucleic Acids Res..

[16] Ineichen B.V., Held U., Salanti G., Macleod M.R., Wever K.E. (2024). Systematic review and meta-analysis of preclinical studies. Nat. Rev. Methods Primers.

[17] Kim M.B., Lee Y., Bae M., Kang H., Pham T.X., Hu S., Lee J.Y., Park Y.K. (2020). Comprehensive characterization of metabolic, inflammatory and fibrotic changes in a mouse model of diet-derived nonalcoholic steatohepatitis. J. Nutr. Biochem..

[18] Ghallab A., Myllys M., Friebel A., Duda J., Edlund K., Halilbasic E., Vucur M., Hobloss Z., Brackhagen L., Begher-Tibbe B. (2021). Spatio-Temporal Multiscale Analysis of Western Diet-Fed Mice Reveals a Translationally Relevant Sequence of Events during NAFLD Progression. Cells.

[19] Fu R., Zhang F., Fu J., Li Y., Zhu X., Ding Q., Zhu Y.Z. (2025). Beyond the liver: Targeting the hepatic microenvironment and multi-organ networks for innovative MASH therapy. Metab. Target Organ Damage.

[20] Dey P. (2025). The Gut–Spleen Axis in Liver Disease: Mechanistic Insights Into Metabolism, Metabolome, and Microbiome Interactions. FASEB J..

[21] Pantano L., Agyapong G., Shen Y., Zhuo Z., Fernandez-Albert F., Rust W., Knebel D., Hill J., Boustany-Kari C.M., Doerner J.F. (2021). Molecular characterization and cell type composition deconvolution of fibrosis in NAFLD. Sci. Rep..

[22] Frankish A., Diekhans M., Jungreis I., Lagarde J., Loveland J.E., Mudge J.M., Sisu C., Wright J.C., Armstrong J., Barnes I. (2021). GENCODE 2021. Nucleic Acids Res..

[23] Dobin A., Davis C.A., Schlesinger F., Drenkow J., Zaleski C., Jha S., Batut P., Chaisson M., Gingeras T.R. (2013). STAR: Ultrafast universal RNA-seq aligner. Bioinformatics.

[24] Liao Y., Smyth G.K., Shi W. (2014). featureCounts: An efficient general purpose program for assigning sequence reads to genomic features. Bioinformatics.

[25] Love M.I., Huber W., Anders S. (2014). Moderated estimation of fold change and dispersion for RNA-seq data with DESeq2. Genome Biol..

[26] Viechtbauer W. (2010). Conducting meta-analyses in R with the metafor package. J. Stat. Softw..

[27] Liberzon A., Birger C., Thorvaldsdóttir H., Ghandi M., Mesirov J.P., Tamayo P. (2015). The Molecular Signatures Database (MSigDB) hallmark gene set collection. Cell Syst..

[28] Subramanian A., Tamayo P., Mootha V.K., Mukherjee S., Ebert B.L., Gillette M.A., Paulovich A., Pomeroy S.L., Golub T.R., Lander E.S. (2005). Gene set enrichment analysis: A knowledge-based approach for interpreting genome-wide expression profiles. Proc. Natl. Acad. Sci. USA.

[29] Korotkevich G., Sukhov V., Budin N., Shpak B., Artyomov M.N., Sergushichev A. (2021). Fast gene set enrichment analysis. bioRxiv.

[30] Davis S., Meltzer P.S. (2007). GEOquery: A bridge between the Gene Expression Omnibus (GEO) and BioConductor. Bioinformatics.

[31] Robinson M.D., McCarthy D.J., Smyth G.K. (2010). edgeR: A Bioconductor package for differential expression analysis of digital gene expression data. Bioinformatics.

[32] Ritchie M.E., Phipson B., Wu D., Hu Y., Law C.W., Shi W., Smyth G.K. (2015). Limma powers differential expression analyses for RNA-sequencing and microarray studies. Nucleic Acids Res..

[33] Robinson M.D., Oshlack A. (2010). A scaling normalization method for differential expression analysis of RNA-seq data. Genome Biol..

[34] Law C.W., Chen Y., Shi W., Smyth G.K. (2014). voom: Precision weights unlock linear model analysis tools for RNA-seq read counts. Genome Biol..

[35] Dyer S.C., Austine-Orimoloye O., Azov A.G., Barba M., Barnes I., Barrera-Enriquez V.P., Becker A., Bennett R., Beracochea M., Berry A. (2025). Ensembl 2025. Nucleic Acids Res..

